# Effectiveness of focused extracorporeal shock wave versus manual therapy in postpartum patients with sacroiliac joint dysfunction: a prospective clinical trial

**DOI:** 10.1186/s13018-023-04491-0

**Published:** 2024-01-03

**Authors:** Kuai-ling Tan, Rong Wang, Jiao-jiao Liu, Yue Peng, Huan Li, Cui-ying Li

**Affiliations:** 1 Pelvic Floor and Postpartum Rehabilitation Center, Changsha Maternal and Child Health Care Hospital, Chengnan Dong Lu, Yuhua District, Changsha, 410007 Hunan China; 2 Department of Gynecology II, Changsha Maternal and Child Health Care Hospital, Changsha, 410007 Hunan China; 3 Product R&D, Shenzhen Creative Industry Co., Ltd., Shenzhen, 518055 Guangdong China

**Keywords:** Sacroiliac joint dysfunction, Focused extracorporeal shock, Pain, Oswestry disability index score

## Abstract

**Objective:**

To investigate the effectiveness of focused extracorporeal shock wave therapy (FESWT) in treating postpartum sacroiliac joint (SIJ) dysfunction.

**Methods:**

A total of 90 patients with SIJ dysfunction were included and randomly assigned to FESWT, manual therapy (MT), or combination therapy (CT) groups. Pain intensity and Oswestry Disability Index (ODI) score were measured upon admission, after 1 and 2 weeks of treatments. The treatment efficacy and adverse events of each group were also assessed.

**Results:**

There were no significant differences among three groups regarding clinical data, pain intensity, and ODI score on admission (all *P* > 0.05). After 1 week of treatment, FESWT exhibited similar pain intensity and lower ODI score (*P* < 0.001) compared to MT. After 2 weeks of treatment, the pain and ODI in FESWT were similar with MT. The pain in CT was lower than MT after 1 week, but lower than FESWT after 2 weeks. Furthermore, we identified interaction effects between treatment method and duration in relation to pain intensity (*F*_group*time_ = 5.352, *P* = 0.001) and ODI score (*F*_group*time_ = 5.902, *P* < 0.001). FESWT group exhibited the highest improvement rate of 66.7%, while CT group achieved the highest cure rate of 73.3%. No adverse events were observed in any of the patients during 2 months follow-up period.

**Conclusions:**

Compared to MT, FESWT mainly reduced the ODI score rather than pain after 1 week of treatment. After 2 weeks, the effect of FESWT in relieving the pain was inferior to the MT.

## Introduction

The sacroiliac joints (SIJ), being the largest axial joints, are situated in the pelvis and connect the iliac bones to the sacrum. Pain experienced in the SIJ, hip joint, lumbar spine, and visceral region can result in pain in SIJ region. The presence of pain and stiffness in the SIJ region is commonly associated with SIJ dysfunction. Risk factors for SIJ dysfunction encompass pregnancy, obesity, prolonged periods of sitting, leg length discrepancy, excessive physical activity, direct trauma, systemic inflammatory conditions, and degenerative joint diseases [1]. Both hypo and hyper-mobile SIJ are considered abnormal motion patterns. Individuals with pelvic fractures and a sedentary lifestyle commonly experience hypo-mobility and joint fixation, potentially increasing their susceptibility to SIJ dysfunction. Conversely, during pregnancy, the SIJ heightened mobility due to the sex hormones and relaxin, which promote ligamentous laxity. Various biomechanical factors contribute to SIJ dysfunction in pregnancy, including augmented weight, alterations in posture, heightened abdominal and intrauterine pressure, and laxity on the spine and pelvic structures [2]. Furthermore, a previous study has also indicated the presence of SIJ dysfunction resulting from symphysiolysis in postpartum women [2]. It has been observed that 44.8% of patients with SIJ dysfunction had developed it as a consequence of childbirth [3].

Initially, conservative management is recommended as the primary treatment for SIJ dysfunction. However, if conservative management proves ineffective within a span of 6 weeks, alternative approaches such as intra-articular injections, peri-articular injections, or nerve blocks may be considered [4]. Additionally, the efficacy of manual therapy and exercise therapy in addressing SIJ syndrome has been extensively explored in various studies [5–7]. Recently, extracorporeal shock wave therapy (ESWT) has been applied for the treatment of temporomandibular disorders [8] and plantar fasciitis (treated on myofascial points) [9]. It has been recognized as a biological modulator, capable of inducing the differentiation of mesenchymal stem cells, promoting neovascularization, and facilitating the release of angiogenetic factors [10]. Currently, only one study evaluated the efficacy of using ESWT to treating SIJ pain [11], which highlighted that ESWT represents a potential therapeutic option for decreasing SIJ pain. However, the effectiveness of focused ESWT in treating SIJ dysfunction in postpartum woman remains unrevealed.

In this study, we conducted a prospective randomized controlled trial to explore the therapeutic effect of focused ESWT on SIJ dysfunction syndrome in postpartum women, and to evaluate the efficacy of focused ESWT on pain relief and SIJ function recovery. This study contributes to provide reference value for the treatment of SIJ disorder in postpartum women and improve their quality of life.

## Methods

### Participants

A prospective randomized controlled trial was conducted on postpartum women diagnosed with postpartum SIJ dysfunction between July 2022 and December 2022 at our hospital. This study received approval by the Institutional Review Board of our hospital. Informed consent was obtained from all participants.

The inclusion criteria for this study were as follows: (1) meeting specific diagnostic criteria for SIJ dysfunction; (2) primiparas with a singleton pregnancy within 1 year after delivery, aged between 20 and 35 years; (3) presence of unilateral SIJ dysfunction; (4) according to the symptoms of lower lumbosacral pain and the local physical examination of sacroiliac joint, the pain in SIJ was suggested; (5) written the informed consent.

The following patients were excluded from the study: (1) those experiencing pain in the waist and legs due to alternative etiologies; (2) pregnant individuals; (3) individuals with sacroiliac joint sprain characterized by symmetrical bone marks and no abnormalities observed in X-ray images; (4) individuals with tuberculosis affecting the sacroiliac joint or spine; (5) individuals with tumors; (6) individuals with fractures; and (7) individuals with ankylosing spondylitis.

The diagnostic criteria of SIJ dysfunction in this study referred to the European guidelines for the diagnosis and treatment of pelvic girdle pain in 2008, including (1) have a history of trauma or maternity; (2) pain occurs in unilateral or bilateral sacroiliac joint and upper hip, and pain aggravates when turn over; (3) muscle spasm around SIJ, limited activity of lower limbs, inability to sit for a long time; (4) SIJ of the affected side is swollen and appears bulge than healthy side; (5) pelvic compression and separation tests, “4” test, and one-foot standing test are all positive; (6) X-ray examination of pelvis shows that the SIJ space of the affected side is slightly widened, the articular surface is disordered, and the pubic symphysis moves up and down slightly.

### Randomization

In this study, covariate adaptive randomization (CAR) was performed to assign patients to 3 groups. The age, course, and pain intensity on admission were considered as covariates. We conducted the allocation concealment and detailed patient assignment was concealed from the investigators. The patients were aware of their treatment, but were blinded to the treatment details of other groups. Further, we carried out the physical examination on patients and collected their baseline clinical information including age, course, site of unilateral pain (left or right), pain during pregnancy (yes/no), breastfeed their baby after delivery (yes/no), and pain intensity.

### Interventions

This study included postpartum women diagnosed with SIJ dysfunction within 1 year after delivery, who were then randomly allocated into three groups. The first group received focused extracorporeal shock wave therapy (FESWT), the second group underwent the manual therapy (MT), and the third group received a combination therapy (CT) consisting of FESWT combined with MT. All patients received a course of therapy, and a course consisted of 5 times of treatment (20 min/time). The treatment was conducted every 2 days, and a course of therapy needed 2 weeks.

1. Interventions in FESWT group involved the utilization of a cutting-edge electromagnetic focused shock wave therapy device (FS10Pro, Shenzhen Creative Industry Co., Ltd., China). The energy flux density (EFD) of the device was set at 0.0298 mJ/mm^2^. The focal range of focused ESWT device: radial ≤  ± 6 mm, axial ≥ 60 mm. The treatment was administered every 2 days, and a course of therapy lasted for 2 weeks. For the shock wave therapy, 4 bone markers, namely the anterior superior iliac spine, posterior superior iliac spine, iliopubic eminence, and ischial tuberosity, were chose to receive the shock. Each bone marker is symmetrical left and right. Hence, a total of 8 points were administrated the shock. The 500 shocks were set for each bone marker point, and a total of 4000 shocks were finally applied.

For the treatment plan, it was determined according to the metabolism rate of ESWT metabolite. The ESWT has been found to upregulate the expression of collagen, initiate cell proliferation in healthy tenocytes, and positively affect the metabolism of tendons. It can also be able to induce positive modulation of tendon-specific marker expression and release of anti-inflammatory cytokines [12]. In our clinical practice, we found that these effects were weakened after 2–3 days of FESWT treatment. Therefore, we finally determined the interval time (2 days) of ESWT treatment for patients with sacroiliac joint dysfunction. In addition, during the clinical treatment, we found that the SIJ dysfunction can be obviously improved after half a month. Therefore, we set the course of ESWT therapy as 2 weeks.

For the site selection of FESWT administration, 4 bone markers (anterior superior iliac spine, posterior superior iliac spine, iliopubic eminence, and ischial tuberosity) were treated. Muscles involved in SIJ dysfunction include erector spinae, multifidus muscle, gluteus maximus, gluteus medius, iliopsoas-costal muscle, lateral external oblique muscle, and internal oblique muscle [13, 14]. The myofascial trigger points of these muscles were located around the above bone markers. Eftekharsadat et al. found that extracorporeal shock wave therapy on inferior trigger points in the quadratus lumborum muscle can achieve a favorable reduction in pain and ODI score [15]. Monclús et al. also suggested that shock waves seem to be a suitable treatment for myofascial trigger points [16]. In our clinical practice, we found that the treatment effect was more favorable and sacroiliac joint dysfunction was significantly improved when the myofascial trigger points were treated. For example, the trigger point of gluteus maximus is around the ischial tubercle. When we treated the hip soft tissue with shock wave in clinic, the curative effect was not significant, but the pain was relieved significantly by treating the bone marker of ischial tubercle. Therefore, in this study, 4 bone markers as the trigger points of sacroiliac joint dysfunction were finally selected for FESWT treatment.

(2) Interventions in MT group were as following. Patient assumed a prone position, during which the operator administered a massage and applied pressure to the disordered lumbosacral muscles (latissimus dorsi, erector spinae, multifidus, gluteus maximus, gluteus medius, iliopsoas, lateral external oblique muscle, and internal oblique muscle) for approximately 10 min. When cord-like painful nodule at SIJ was touched, the operator flipped the nodule with thumb and gently pushed it obliquely upward or downward until the nodule reduced or disappeared. Subsequently, the patient transitioned to a supine position, and their lower limb was straightened and subjected to a ringlike shaking motion 6–7 times. The patient's affected side was then positioned beneath the operator's arm, and they received forward-bending traction for 1 min, involving flexion of the knees and hip, as well as adduction of the hip joint. Finally, the operator positioned the patient in lateral decubitus on the healthy side and instructed them to flex their knees and hips. The operator then applied pressure and movement to the affected side of the sacroiliac joint (SIJ), followed by straightening the affected extremity. This treatment process was repeated 3–5 times. Subsequently, the patients were instructed to assume the prone position, and various assessments, including measuring the height of the posterior superior iliac spine, evaluating painful cord-like nodules, conducting the "4" test, and performing point of care testing, were performed to assess the degree of SIJ dysfunction.
(3) Patients in the CT group were received the FESWT and MT simultaneously according to the above interventions.

### Outcomes

The main outcome included pain intensity and Oswestry disability index (ODI) score [17, 18]. The pain condition and ODI score were recorded at admission, after 1 week, and 2 weeks of treatment for all patients. Pain intensity was assessed using the visual analogue scale (VAS) score, with 0 indicating no pain; less than 3 indicating mild pain within an acceptable range; 4–6 indicating severe pain affecting daily life; and 7–10 indicating intense pain that is intolerable. The disability of SIJ was evaluated using the ODI score, which consisted of 9 terms graded on a scale of 0–5. The total ODI score = [sum of 9 terms scores /45] * 100%. A higher ODI score indicated a greater degree of functional impairment.

After the completion of the two-week treatment period, we assessed the overall clinical efficacy within three distinct groups. The clinical efficacy was categorized into three degrees, namely invalid, improved, and cured. An invalid outcome was characterized by the presence of referred pain in the lower extremities caused by SIJ disease, as well as a lack of improvement or even exacerbation or deterioration in the movement function of the lower extremities and sacroiliac joint. Conversely, an improved outcome indicated a reduction in referred pain in the lower extremities caused by SIJ disease, as well as an improvement in the movement function of the lower extremities and sacroiliac joint when compared to the previous state.

When the treatment was finished after 2 weeks, we assessed the overall clinical efficacy among 3 groups. The clinical efficacy was classified to 3 degrees (invalid, improved, cured). Invalid outcome indicates that (1) referred pain exits in lower extremities caused by SIJ disease; (2) the movement function of lower extremities and sacroiliac joint is not improved or even aggravated or deteriorated. Improved outcome indicated that (1) referred pain in lower extremities caused by SIJ disease is reduced; the movement function of lower extremities and sacroiliac joint is improved compared with that before treatment. The presence of a cured outcome suggests that the manifestation of referred pain in the lower extremities, resulting from sacroiliac joint (SIJ) disease, is overlooked, while the functional mobility of both the lower extremities and the sacroiliac joint is normal. Both improved and cured outcomes fall within the realm of effective treatment. The adverse events experienced by all patients were monitored for a duration of two months following the completion of the treatment.

### Statistical analysis

The parameters for calculating the sample size in this study include *α*, *β*, and the effective rate of each group. The effective rate in each group = number (improved + cured) /all patients. There was no study reported the effectiveness of ESWT in treating the SIJ dysfunction in postpartum woman until now. Therefore, to obtain the proper sample size of this study, we performed the preliminary investigation to initially estimate the effective rate of each group. The initial effective rate in preliminary investigation was 80% in MT group, 85% in FESWT group, and 95% in combination group. According to the parameters including *α* = 0.05, 1 − *β* = 0.95, and estimated effective rate of each group, the sample size of 84 for the final study was calculated by G*Power software with the effect size of 0.78. In addition, considering the missing rate and data deficiency into consideration, the approximately 20% of patients was added to the calculated sample size. Finally, a total of 99 patients were enrolled in this study and 33 patients in each group were required.

The data were analyzed using the SPSS software. The data of age, course, weight, weight gain during pregnancy, pain intensity, and ODI score were presented as means and standard error, and their differences among 3 groups were compared using the one-way ANOVA. Regarding the pain intensity and ODI score, we also conducted the post hoc comparisons after ANOVA with Bonferroni method and the corrected *P* < 0.016 was considered statistically significant. In addition, the comprehensive effects of treatment methods and time on the pain intensity and ODI score were measured using the repeated analysis of measurement variance. The qualitative data including location of pain, pain status during pregnancy, postpartum breast feeding, and effective rates were presented as frequency, and the differences among 3 groups were analyzed by χ^2^ and Kruskal–Wallis test. The *P* value less than 0.05 was considered statistically significant.

## Results

### The baseline of participants

Out of the 99 estimated samples, a total of 90 patients diagnosed with SIJ dysfunction were ultimately selected for participation in this study. These patients were then randomly assigned to three distinct groups, each receiving the appropriate treatment. Upon initial assessment (Table 1), no statistical difference was observed among the three groups in terms of age (*P* = 0.92), duration of symptoms (*P* = 0.13), weight gain during pregnancy (*P* = 0.40), location of pain (*P* = 0.87), pain experienced during pregnancy (*P* = 0.12), and postpartum breastfeeding (*P* = 0.82).

**Table 1:** Basic characteristics of patients with SIJ dysfunction among 3 groups

Variables		Focused ESWT	Manual therapy	Combination therapy	χ^2^/t	*P*
Age (years)^a^		30.73 ± 0.72	30.56 ± 0.73	30.33 ± 0.74	0.07^*^	0.92
Course (months)^a^		3.90 ± 0.34	3.75 ± 0.26	3.10 ± 0.26	2.07^*^	0.13
Weight gain during pregnancy/Kg^a^		13.90 ± 0.62	12.65 ± 0.67	13.10 ± 0.68	0.91^*^	0.40
Location of pain^b^	Left	16	16	16	1.22^#^	0.87
	Right	7	11	8		
	Bilateral	7	5	6		
Pain during pregnancy^b^	No	16	18	23	4.14^#^	0.12
	Yes	14	14	7		
Postpartum breast feeding^b^	No	5	7	5	0.37^#^	0.82
	Yes	25	25	25		

^a^The data were presented as means and standard error

^b^The data were presented as frequency

*The data were analyzed by *t*-test; #, the data was analyzed by χ^2^ test

### Treatment effects comparison among 3 groups

We initially assessed the clinical effectiveness of 3 groups at different time points. As shown in Table 2, there were no significant differences in pain intensity (*P* = 0.985) and ODI score (*P* = 0.360) among 3 groups on admission. After 1 week of treatment, both the pain intensity (*P* = 0.001) and ODI score (*P* < 0.001) showed differences among 3 groups. Followed by pairwise comparisons (Table 3), the detailed differences of pain intensity (*P* = 0.001) and ODI score (*P* < 0.001) were found between manual therapy and combination therapy groups. In addition, the difference of ODI score can also be found between focused ESWT and manual therapy (*P* < 0.001).

**Table 2:** Comparison of clinical efficacy among 3 groups by one-way ANOVA

Variables	Focused ESWT	Manual therapy	Combination therapy	*P*
*Pain intensity*				
On admission	5.233 ± 0.156	5.233 ± 0.149	5.200 ± 0.168	0.985
After 1 week	2.700 ± 0.145	3.167 ± 0.136	2.433 ± 0.124	0.001
After 2 weeks	1.200 ± 0.181	0.967 ± 0.188	0.367 ± 0.122	0.002
*ODI score*				
On admission	38.600 ± 0.650	38.670 ± 0.750	37.470 ± 0.560	0.360
After 1 week	24.867 ± 1.285	32.467 ± 0.933	24.267 ± 1.303	< 0.001
After 2 weeks	9.400 ± 1.534	10.267 ± 2.006	3.800 ± 1.287	0.013

**Table 3:** The P values for the post hoc comparisons on pain intensity and ODI score

Groups	On admission	After 1 week	After 2 weeks
*Pain intensity*			
FESWT-MT	0.982	0.050	0.977
FESWT-CT	0.891	0.502	0.002
MT-CT	0.798	0.001	0.038
*ODI score*			
FESWT-MT	0.979	< 0.001	0.970
FESWT-CT	0.690	0.975	0.053
MT-CT	0.612	< 0.001	0.019

*FESWT* Focused extracorporeal shock wave therapy; *MT* Manual therapy; *CT* Combination therapy of FESWT and MT. The post hoc comparisons were performed with Bonferroni method and corrected *P* < 0.016 was considered statistically significant

The differences of pain intensity and ODI score were also observed among 3 groups after 2 weeks of treatment (Table 2, *P* = 0.013). Followed by pairwise comparisons, the pain intensity in focused ESWT was higher compared with combination therapy (*P* = 0.002). The ODI showed no difference between any two groups even though the difference of ODI was significant among three groups.

**Fig. 1:**
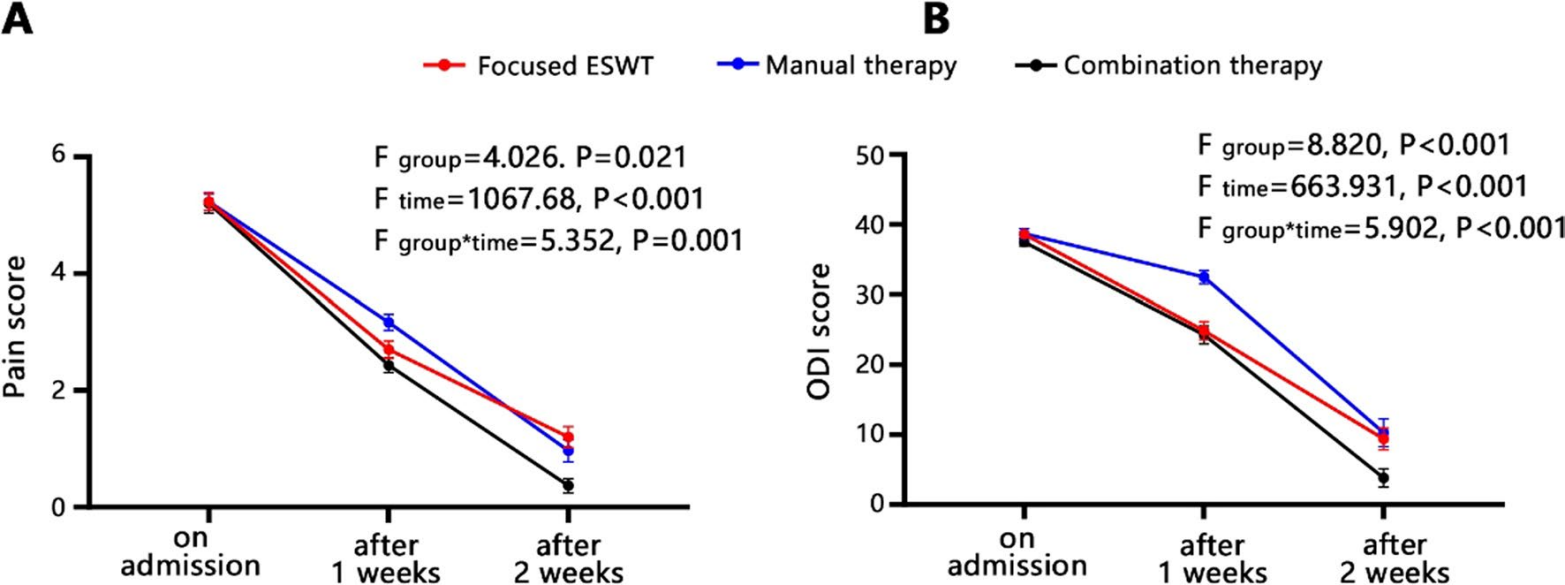
Repeated analysis of measurement variance for assessing the clinical efficacy among 3 groups. **A** Pain intensity. **B** ODI score

We further conducted repeated analysis of measurement variance to evaluate the clinical efficacy among 3 groups (Fig. 1A and B). The χ^2^ value of Mauchly’s test of sphericity for pain intensity and ODI score was found to be 11.045 (*P* = 0.004) and 16.132 (*P* < 0.001), respectively, which did not conform with the sphericity hypothesis. Therefore, the comparison of effects within-subjects was analyzed using the adjusted Greenhouse–Geisser method. The results revealed that the P values for the test of between-subjects effects on pain intensity (*P* = 0.021) and ODI score (*P* < 0.001) were both below the threshold of 0.05, indicating their difference among 3 treatment methods. In addition, the P values for various time points and interaction effects concerning pain intensity and ODI score were also found to be less than 0.05, indicating a significant variation in clinical efficacy among 3 groups at different time points. The impact of treatment methods on pain and ODI scores exhibited temporal variability.

The mean treating times for the focused ESWT, manual therapy, and combination therapy groups were 4.76, 4.67, and 4.91 times, respectively. There was no statistical difference in treating times among the 3 groups. A number of patients with milder symptoms in all three groups experienced favorable efficacy after three treatment sessions. Additionally, we assessed the overall effectiveness rate after two weeks of treatment. Table 4 demonstrates that none of the patients in any of the three groups were deemed ineffective. However, there was a significant difference in the effectiveness rates among the three groups (*P* = 0.008). The focused Extracorporeal Shock Wave Therapy (ESWT) demonstrated the most notable rate of improvement (66.7%), while combination therapy exhibited the highest rate of successful treatment (73.3%). Across all included samples, no significant adverse events, side effects, or severe complications, such as hematomas or abnormal musculoskeletal events, were reported within each group after a 2-month follow-up period. A total of 9 samples withdrew from the study due to reasons that remain unknown or unrelated to the interventions.

**Table 4:** Comparison of clinical efficacy among 3 groups after 4 weeks of treatment

	N	Ineffective	Improved	cured	*P*
Focused ESWT	30	0	20 (66.7%)	10 (33.3%)	0.008
Manual therapy	30	0	15 (50%)	15 (50%)	
Combination therapy	30	0	8 (26.7%)	22 (73.3%)	

The data were analyzed by Kruskal–Wallis test

## Discussion

The objective of this study was to investigate the therapeutic effects of focused extracorporeal shockwave therapy (ESWT) on sacroiliac joint (SIJ) dysfunction in postpartum women. Our findings indicated that focused ESWT yielded significant reduction of ODI score compared to manual therapy after 1 week of treatment. However, it did not significantly reduce the pain intensity after 2 weeks. To the best of our knowledge, this study is the first to emphasize the importance of focused ESWT in the treatment of SIJ dysfunction in postpartum women.

The prevalence of chronic pain postpartum is high among women, potentially attributed to the structural changes in the pelvis that occur during pregnancy and postpartum [19]. This can result in the experience of sacroiliac joint pain. Specifically, women with asymmetrical SIJ laxity during pregnancy are at a three-fold increased risk for developing postpartum pain [20]. The asymmetrical SIJ laxity disrupts the balance of forces exerted on the SIJ, consequently leading to SIJ dysfunction that may persist into the postpartum period following pregnancy [21]. The previous study presented that 36% of women showed the SIJ pain during pregnancy and 23% of those patients had continual pain postpartum. The prevalence of SIJ disorders among women has emerged as a significant health concern. Notably, the incidence of SIJ misalignment in young women surpasses that in males, owing to inherent biomechanical disparities in the SIJ and the greater loads, stresses, and increased SIJ mobility [2]. However, it is crucial to acknowledge that postpartum SIJ dysfunction often goes underestimated, as women tend to seek medical attention only when the discomfort significantly hampers their daily activities. Consequently, the effective management of postpartum SIJ dysfunction assumes paramount importance in enhancing the overall quality of life for these women.

In this study, we found that focused ESWT significantly improved the SIJ dysfunction of postpartum woman, which can be regarded as an alternative therapy. Few studies investigated the effects of focused ESWT on SIJ dysfunction in postpartum woman. It was also reported that the influence of shock wave energy or dose on efficacy remains debatable; for example, high-energy ESWT is suitable for treating calcified tendinitis [22] whereas low dose is suitable for pain reduction for plantar fasciitis [23]. It follows that the shock wave energy may be vary from disease to disease. In our study, the focused shock wave therapy device was used to release the pain of patients with sacroiliac joint dysfunction, and low-energy ESWT may be more suitable. The previous study has reported that an EFD less than 0.2 mJ/mm^2^ was identified as the optimal energy for focused ESWT for tissue regeneration [24]. However, when the energy was applied into the patients with sacroiliac joint dysfunction, the patients cannot stand this EFD 0.2 mJ/mm^2^. Therefore, we made a number of readjustments to the proper EFD, finding that 0.03–0.07 mJ/mm^2^ EFD can achieve a favorable efficacy for the pain reduction in patients with sacroiliac joint dysfunction. Finally, the energy of the focused ESWT used in this study was set as 0.0298 mJ/mm^2^, suggesting that lower energy level of focused ESWT was enough to achieve a more favorable efficacy for the treatment of postpartum SIJ dysfunction. This study further supported the obvious differences of EFD value in different diseases.

In addition, the intervention period of focused ESWT may influence its efficacy [25]. In the present study, it was observed that focused ESWT administered after 1 week yielded a significant improvement in ODI reduction when compared to manual therapy. This finding suggests that focused ESWT exhibits superior efficacy for releasing the SIJ dysfunction. Furthermore, the effectiveness of focused ESWT on ODI after two weeks was found to be comparable to that of manual therapy.

Our study provides valuable information regarding the ESWT efficacy in treating SIJ dysfunction in postpartum woman, but several limitations should be acknowledged. Our findings may be limited due to the small sample size and lack of long-term follow-up. This study just can be regarded as a short-term experimental trial. In addition, it was a single center study. Further studies are required with larger samples and longer follow-up time to validate our results. Patient stratification is also needed to optimize the patient conditions for better treatment outcomes.

## Conclusion

This study aimed to investigate the treatment efficacy of focused ESWT for releasing postpartum SIJ dysfunction and reducing pain. Our findings revealed that focused ESWT demonstrated a significantly superior effect in reducing ODI score after one week of treatment compared to manual therapy. After two weeks of treatment, both therapies exhibited similar efficacy on reducing ODI score. Comparatively, the combination therapy (ESWT + manual therapy) had more favorable treatment effect than the single treatment method. No adverse events were reported during the 2 months follow-up period. Our results suggests that focused ESWT can be considered an effective therapeutic approach for postpartum SIJ dysfunction. There is a need to explore the long-term effects of focused ESWT on SIJ dysfunction in postpartum women.

## Data Availability

The datasets generated during and/or analyzed during the current study are available from the corresponding author on reasonable request.
